# Enhancing Fused Deposition Modeling Precision with Serial Communication-Driven Closed-Loop Control and Image Analysis for Fault Diagnosis-Correction

**DOI:** 10.3390/ma17071459

**Published:** 2024-03-22

**Authors:** Saeed Behseresht, Allen Love, Omar Alejandro Valdez Pastrana, Young Ho Park

**Affiliations:** Department of Mechanical and Aerospace Engineering, New Mexico State University, Las Cruces, NM 88003, USA; behsaeed@nmsu.edu (S.B.); alove@nmsu.edu (A.L.); pastrana@nmsu.edu (O.A.V.P.)

**Keywords:** additive manufacturing, fused deposition modeling, closed-loop quality control system

## Abstract

Additive manufacturing (AM) also commonly known as 3D printing is an advanced technique for manufacturing complex three-dimensional (3D) parts by depositing raw material layer by layer. Various sub-categories of additive manufacturing exist including directed energy deposition (DED), powder bed fusion (PBF), and fused deposition modeling (FDM). FDM has gained widespread adoption as a popular method for manufacturing 3D parts, even for heavy-duty industrial applications. However, challenges remain, particularly regarding part quality. Print parameters such as print speed, nozzle temperature, and flow rate can significantly impact the final product’s quality. To address this, implementing a closed-loop quality control system is essential. This system consistently monitors part surface quality during printing and adjusts print parameters upon defect detection. In this study, we propose a simple yet effective image analysis-based closed-loop control system, utilizing serial communication and Python v3.12, a widely accessible software platform. The system’s accuracy and robustness are evaluated, demonstrating its effectiveness in ensuring FDM-printed part quality. Notably, this control system offers superior speed in restoring part quality to normal upon defect detection and is easily implementable on commercially available FDM 3D printers, fostering decentralized quality manufacturing.

## 1. Introduction

Additive manufacturing (AM), alternatively referred to as 3D printing, solid freeform fabrication, or rapid prototyping, stands as a revolutionary manufacturing method poised to transform the landscape of various industries in the future [1]. In contrast to traditional manufacturing methods, where products are shaped through pressure application or subtracting material from a solid, additive manufacturing operates by depositing material at specified locations using computer-aided design (CAD) and printers. This process involves adding layers of material to create solid components, allowing the cost-effective and easy production of complex geometries [2]. A variety of materials, such as plastics, metals, and ceramics, are employed in the additive manufacturing process [3,4]. Certain additive manufacturing techniques utilize chemical adhesion or crosslinking processes to connect layers at room temperature. Examples of such methods include vat photopolymerization, material jetting, binder jetting, and direct-write. Conversely, alternative methods such as fused deposition modeling (FDM), directed energy deposition (DED), powder bed fusion (PBF), and thermoplastic material extrusion rely on heat to liquefy and merge new material layers into the previously created one [5].

However, ensuring the quality of additive manufacturing (AM) parts poses an ongoing challenge. Despite the remarkable capabilities of AM processes, the intricacies of consistently achieving high-quality outcomes persist as a significant concern. Factors such as material variations, process parameters, and post-processing techniques contribute to the complexity of the challenge. Achieving uniformity and reliability in AM parts becomes crucial, especially when considering their application in critical industries such as aerospace, healthcare, and automotive [6]. Among the group of 3D printing techniques available, fused deposition modeling (FDM) stands out as one of the most prevalent, versatile, and cost-effective methods. FDM, also recognized as fused filament fabrication (FFF), involves the use of a continuous filament made of either pure or composite thermoplastic polymers which has proved much more cost effective compared to various traditional techniques [7].

In the FDM process, the filament undergoes initial heating to a semi-liquid state before being extruded, with the nozzle following a predetermined printing path. Following deposition, thermal energy dissipates through conduction within and between filaments as well as through convection and radiation between the filament and the external environment. The heating and cooling phases cause the filament to return to a solid state and exhibit significant thermal gradients that induce residual stresses on the printed component. These stresses can result in part distortions either during the printing process itself or after the component is removed from the building plate [8]. These complexities in the FDM process are accompanied by other issues, including voids, over-fill, and under-fills. Voids, creating empty spaces within the material structure, compromise the overall strength and stability of the printed component. Over-fill and under-fill contribute to inaccuracies in dimensions and geometrical fidelity, impacting the final product’s functionality. Additionally, challenges such as layer misalignment, warping, and inconsistent extrusion make up the list of common defects in FDM. Layer misalignment can result in structural weaknesses, warping distorts the intended shape of the object, and inconsistent extrusion leads to uneven material distribution [9,10]. By addressing this set of issues, high-quality FDM prints can be achieved, ensuring the technology’s efficacy across diverse applications.

Numerous models and algorithms have been proposed in the field to enhance the quality and integrity of FDM printed parts. Wu et al. [11] used acoustic emission sensors to develop an in situ monitoring framework to monitor the machine conditions during fused filament fabrication (FFF). Kousiatza et al. [12] proposed a methodology using fiber Bragg grating sensors to capture temperature and strain distributions of the FFF.

Li et al. [13] proposed a data-driven predictive methodology based on multiple machine learning algorithms for the FDM process to enhance the reliability of the additive manufacturing process in producing high-quality components. The have used temperature and vibration data from multiple sensors including thermocouples, infrared thermometers, and accelerometers to extract the time–frequency feature accordingly. Feng et al. [14] developed a framework utilizing machine learning algorithms to adjust print process parameters, minimizing warpage effects. Kadam et al. [15] proposed a methodology for layer-wise defect detection by integrating several machine learning algorithms and pretrained models. Their combined algorithms were trained offline and implemented online for anomaly detection throughout the print process. The results obtained from their study show that the combination of the SVM and Alexnet algorithm results in maximum accuracy. Kantaros and Piromalis [16] closely examined the 3D printing challenges by testing the effect of key print process parameters on printing results. They assessed resulting printing quality differences and determined the optimal process parameters for achieving the best print quality. Given the critical importance of printed part quality and integrity across diverse applications, the demand for fault detection-correction systems like these is increasingly evident [17].

In this study, we introduce an innovative system designed to detect and correct surface anomalies in the FDM process. Our proposed system has several advantages compared to the existing models. These unique features include fast detection and correction, simplified assembly, and cost-effectiveness, further promoting decentralization within the AM process. We present a comprehensive investigation employing an Ender 5 Plus FDM printer to develop a functional closed-loop control system for monitoring the quality of FDM printed parts. Our proposed image collection system integrates digital microscopes strategically mounted on the printer to capture high resolution images of the printed part’s surface. This system provides an adequate view of the printing process, enhancing our ability to monitor and analyze the quality of each layer. Simultaneously, we implemented a fault correction script and established serial communication between the computer and the 3D printer to have control over g-code commands and printer parameters such as flow rate, temperature, and speed.

## 2. Construction of the Experimental Setup

### 2.1. Implementation Setup

**Figure 1:**
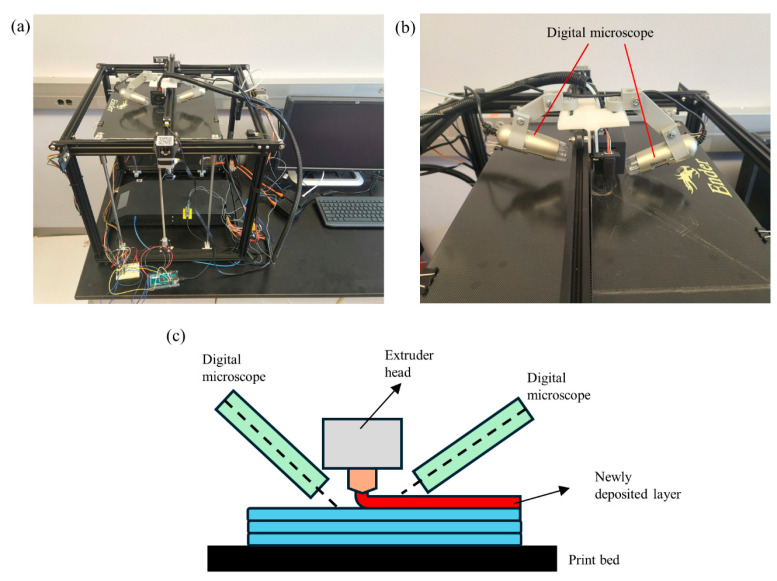
Experimental setup. (**a**) Digital microscopes mounted on extruder head. (**b**) Side view of digital microscopes. (**c**) Image collection schematic.

In this research study, a Creality, Ender 5 Plus printer (Shenzhen, China) [18] was used to conduct the experiments as shown in Figure 1a. The printer has an extruder with 0.4 mm nozzle diameter and uses grey Polylactic Acid (PLA) filament with a diameter of 1.75 mm for the printing process. The proposed image collection system consists of two digital microscopes mounted on opposite sides of the extruder head of the 3D printer, capturing high-resolution images of the printed part’s surface (Figure 1a). The magnification range of the digital microscopes is between 20 and 240 and resolution of captured images is 1280 × 1024 pixels. The images are captured 2 cm away from the surface of the part with magnification of 35. This configuration will improve the monitoring of the entire surface of the printed part around the nozzle. Figure 1b represents the side and highlighted view of the digital microscope assembly, while Figure 1c depicts the schematic of the image collection system.

**Figure 2:**
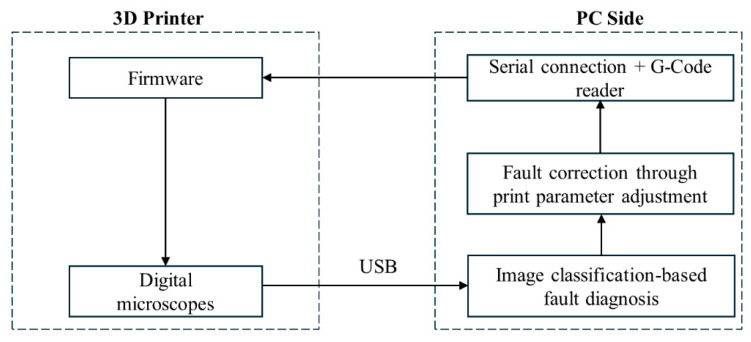
The overall schematic of the fault diagnosis-correction system.

As previously mentioned, the primary objective of this research is to develop a simple yet effective closed-loop control system for monitoring the quality of printed parts in the FDM process. This system is designed to seamlessly integrate with commercially available FDM printers. In this context, the interaction among various components of the system is explained here. Figure 2 illustrates two components on the 3D printer side: the main firmware responsible for overseeing and executing printing operations and the image collection system comprising two digital microscopes. On the PC side, three modules are present: (1) image classification-based fault diagnosis that detects and classifies defects (explained in detail in Section 3.3); (2) fault correction Python script that adjusts print parameters and manipulates g-code commands based on the identified defect; and (3) serial connection facilitating the transmission of unchanged or modified g-code commands to the printer.

Conventionally, two methods were employed to communicate with the 3D printer to initiate a print: SD cards and Octoprint. The SD card allows users to upload the original g-code file into the printer, while Octoprint enables remote transmission of g-code to the printer within the same network through a web interface. In both cases, users are unable to make changes to print parameters.

In this research, a serial communication setup between the computer and the 3D printer was established using a python script. This script reads the g-code file line by line and transmits these commands to the printer for part fabrication. The superiority of serial communication lies in the user’s enhanced control over the connection between the g-code sender (Python v3.12) and the g-code receiver (3D printer). This allows easy manipulation of g-code commands and prompt modification of printer parameters sent to the printer.

Contrary to the conventional approach of embedding a control module in 3D printers, which contributes to their high cost, implementing serial communication provides the flexibility to adjust print parameters on any standard printer available in the market, making them more accessible.

The closed-loop control for fault diagnosis and detection begins by taking a photo of the part’s surface using digital microscope located on the 3D printer side (Figure 2). This photo is then received and classified by an image analysis algorithm on the PC side. Then, based on the identified error type, print parameters are adjusted by submitting new commands or modifying the original commands sent to the printer via serial communication, restoring the surface quality of the print part to normal.

### 2.2. Implementation Cost

This project was carried out with limited resources and can be installed and used on most general 3 axis FDM printers. To demonstrate the concept, we utilized the Creality Ender 5 due to its spacious and simple frame. All brackets and additional printable hardware were designed and produced in-house using this printer. Apart from the 3D printer and a computer capable of running python and modeling software, the essential resources include 2 digital microscopes, a USB-C data-transfer cable, and the PLA filament used for printing the microscope brackets along with other hardware components. Below is a breakdown of the required components needed to replicate this system. Assuming the availability of a computer and printer, the total cost of implementing the proposed Closed-Loop Fault Diagnosis-Correction system (CLFDCS) is less than USD 400 as detailed in Table 1.

The most challenging aspect of implementing this system is to establish how the microscopes will be mounted. To achieve this, a close inspection of the tool head is required. The extrusion heads typically have extra mounting holes or have unnecessary plates and cosmetic structures that can be removed, allowing the microscope brackets to be installed. One specific issue that may change based on the printer’s physical structure and its homing characteristics is the possibility of the microscope’s position obstructing the printer’s frame during homing or the start of a print. To mitigate this issue, the arm was designed to be adjustable, enabling it to be lifted out of the way while the printer processes its initial commands and then positioned correctly for the print.

## 3. Research Approach

The present Closed-Loop Fault Diagnosis-Correction system (CLFDCS) comprises three main modules: the Image Collection Module, Image Analysis-Based Fault Diagnosis Module, and Fault Correction Module. This section provides a comprehensive explanation of all three modules.

### 3.1. Image Collection Module (ICM)

**Figure 3:**
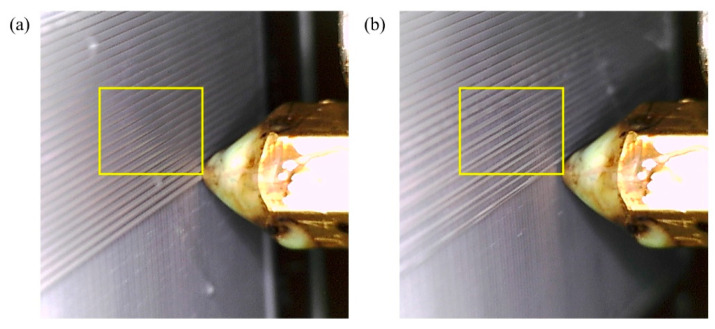
Images taken using digital microscopes and used for Image analysis: (**a**) High quality, (**b**) Low quality. Yellow frame shows the area if interest used for image analysis.

Two USB Dino-Lite digital microscopes [19], featuring adjustable resolution, are mounted on the extruder head, as shown in Figure 1. The images with dimensions 640 × 480 pixels are captured every 30 s and stored in a designated directory on the computer for subsequent use by the image analysis algorithm. Sample photos of the surface of the parts with high and low qualities taken using microscopes are shown in Figure 3a,b, respectively. To effectively capture the surface texture, it is essential to exclude features, such as the nozzle and print bed surface, from the photos. Instead, the focus should only be on the adjacent area around the nozzle (64 × 64 pixels), as highlighted by yellow rectangular areas, for image analysis. This is achieved by cropping the image to small sizes before they are processed for image analysis.

As shown in Figure 1b, two digital microscopes are positioned on opposite sides of the extruder head to ensure effective monitoring of the entire surface around the nozzle. The addition of more microscopes around the nozzle can further address potential blind spots.

### 3.2. Image Analysis-Based Fault Diagnosis Module (IAFDM)

**Figure 4:**
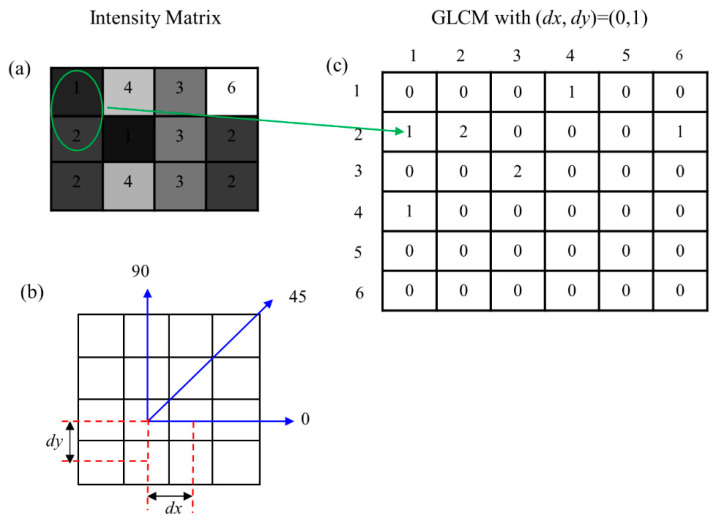
GLCM matrix: (**a**) Intensity matrix, (**b**) Spatial distance used to extract the GLCM matrix, (**c**) Sample GLCM matrix extracted.

From the experiments conducted, it is evident that defects with different severities and surface finishes show distinct textural characteristics [20,21]. Hence, our proposed image classification algorithm incorporates a method for extracting textural features, enhancing the performance and robustness of the image classification process—a crucial component within our CLFDCS. The Grey-Level Co-Occurrence Matrix (GLCM), a classical texture feature extraction technique [22], is employed to achieve this objective. GLCM is a statistical method used in image processing to examine the spatial relationship between pixels. It quantifies the frequency of occurrence of pairs of pixels with specific intensity values at defined distances and orientations within an image. The relative position of this pixel pair is characterized by spatial distance in a Cartesian coordinate system as (*dx*,*dy*) or in a polar coordinate system as (*d*,*θ*). Figure 4b shows spatial distance with three different orientations (*θ* = 0, 45, 90) employed when deriving GLCM.

Various GLCMs can be generated by considering different spatial distances. As an example, Figure 4c demonstrates the calculation of a GLCM with a spatial distance of (*dx*,*dy*) = (0,1) or equivalently (*d*,*θ*) = (1,π/2), based on the provided intensity in Figure 4a. The intensity of each pixel in the given grey-scale image is indicated within the pixels in Figure 4a. The maximum intensity for this grey-scale image is six, corresponding to the brightest pixel, while the minimum intensity is one, corresponding to the darkest pixel. If the greatest intensity is M, then the GLCM becomes a square matrix of dimensions M by M, represented as a matrix of 6 by 6 for the given sample in Figure 4c.

Figure 4 illustrates the procedure of populating GLCM arrays from an intensity matrix. For a given 6-level grayscale image with the dimensions 3 by 4, when setting spatial distance (*dx*,*dy*) = (0,1), the resulting GLCM is a 6 by 6 matrix. G (2,1) within this GLCM is situated in row 2 and column 1, denoting the frequency of occurrence of the pair (2,1) in the intensity matrix. Hence, G (2,1) equals 1, representing the number of times the pair (2,1) is repeated in the intensity matrix.

Depending on the application, GLCM can be employed to extract various statistical features, which are then utilized for textural feature-based image analysis [23,24]. In our research work, we utilize features such as Contrast, Correlation, Energy, and Homogeneity for image analysis [20].

1. Contrast: A measure quantifying the intensity contrast between a pixel and its neighbor, defined as:
  $$\mathrm{Con}=\sum_{i}\sum_{j}\left(i-j)G(i,j\right)$$
2. Correlation: A measure of correlation between a pixel and the neighbor, defined as:
  $$\mathrm{Cor}=\sum_{i}\sum_{j}\frac{\left(i-\mu_{i})(j-\mu_{j})G(i,j\right)}{\sigma_{i}\sigma_{i}}$$
3. Energy: Also known as the angular second moment and defined as:
  $$E=\sum_{i}\sum_{j}G^{2}(i,j)$$
4. Homogeneity: A measure assessing the closeness of the distribution of elements to the diagonal of GLCM, defined as:
  $$H=\sum_{i}\sum_{j}\frac{G(i,j)}{1+{\left(i-j\right)}^{2}}$$

Further details on how to measure these features from GLCM can be found in [25,26]. Once these features are extracted, classification algorithms come into play for the detection of surface defects. This involves utilizing labeled training image data collected during experiments. In our study, we employed LIGHTGBM, a gradient-boosting framework based on decision trees, to increase the efficiency of the model and minimize memory usage. The classifier is trained offline and later utilized to analyze online image data captured by the image collection module discussed in Section 3.1.

### 3.3. Fault Correction Module (FCM)

**Figure 5:**
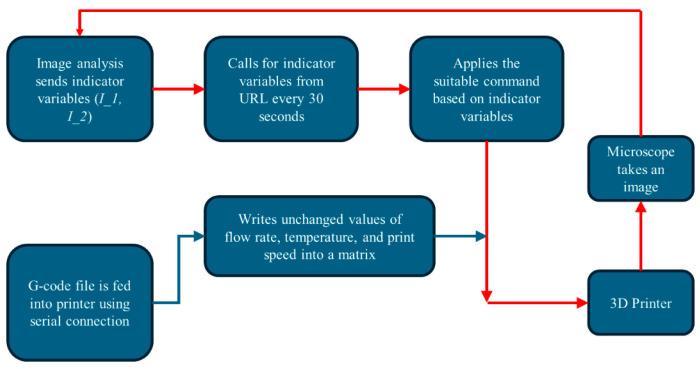
Fault Correction Module framework. Red colored arrows show the closed-loop FCM.

Our Fault Correction Module (FCM) focuses on real-time adjustments to process parameters during printing, with the goal of optimizing print quality by addressing quality issues early in the printing process. It comprises two independent Python scripts and a web URL. One of these scripts hosts a Flask app, acting as a Python web framework that provides users with a specific URL [27]. The second Python script facilitates serial communication (SC) between the PC side and the 3D printer side (Figure 2), performing two main functions: sending G-Code commands to the printer and adjusting G-Code commands that define print parameters as required. The modules communicate using shared values I_1 and I_2, referred to as indicator variables. These variables are assigned to photos based on the types of defects detected by the IAFDM, as outlined in Table 1. Once a defect type is diagnosed by the IAFDM, the corresponding indicator variables are assigned to the photo and transmitted to the URL from the IAFDM to be shared with SC script for adjusting print parameters accordingly. The Fault Correction Module is illustrated schematically in Figure 5.

The IAFDM receives real-time images of the printed part, captured using digital microscopes every 30 s, and assesses them for defects. Subsequently, based on the detected defect type (or non-defect, referred to as “normal”), it assigns the appropriate indicator variables outlined in Table 1 to the image. These assigned indicators are then transmitted as a column matrix over the designated URL.

The SC module initiates with the original G-Code file used for printing. First, the G-Code is converted, line by line, from the “.gcode” file format to an unaltered column matrix represented in “.text” format, encapsulating all instructions necessary for the entire printing process. Next, each line of the G-code is iterated through, and the commands are serially sent to a new column matrix termed the Modified Column Matrix (MCM), which is then forwarded to the printer. Upon detection of a defective image by the IAFDM, updated indicator variables are shared with the SC module.

Following the detection of these events, two types of transformations can be applied to the G-code. If the parameter being adjusted is one that remains fixed throughout the printing process (e.g., temperature or flow rate), a specific command is sent to the printer. For instance, in the case of overfill due to flow rate (I_1 = 1, I_2 = 1), the SC module transmits the command M221 Sxx (xx = current flow rate − *α*) to decrease the flow rate by the designated amount, denoted as *α*. Once adjustments are made, the indicator variables are set, and printing continues with the new parameter settings.

If the parameter being adjusted is one that fluctuates continuously, such as the speed of the extruder head, during the printing process, a multiplier is employed. In G-Code, the extruder head’s speed is controlled by a command starting with the letter F, followed by an integer representing the speed in millimeters per minute. Initially, we utilize a base multiplier of 1, which is then multiplied by the integer value before transmitting the command to the MCM for regular printing operations. Upon detection of a defect caused by excessive printing speed, this multiplier is reduced (e.g., to 0.9), and the remaining G-Code is modified before transmission to the MCM and subsequently to the printer. For instance, if underfill due to high nozzle speed is identified, the multiplier would change from its optimal value of 1 to, for example, 0.85, resulting in a 15% reduction in nozzle movement speed to alleviate underfill by reducing the print speed.

Table 2 lists the defect types, their corresponding indicator variables, and the actions to mitigate them. Note that “current value” indicates the initial print parameter value, such as temperature in Celsius, with subsequent values altered by the FCM.

## 4. Case Studies

This section evaluates the accuracy and reliability of our CLFDCS. As previously discussed, fused deposition modeling presents various sources of error, including extremely low or high extrusion temperatures, inconsistent extrusion rates, and more [28,29,30]. Here in this section, we intentionally generate surface defects by deliberately selecting incorrect print parameters such as temperature, extrusion rate, and printing speed. Then we assess the effectiveness of our CLFDCS in correcting these surface quality abnormalities.

### 4.1. Temperature

**Figure 6:**
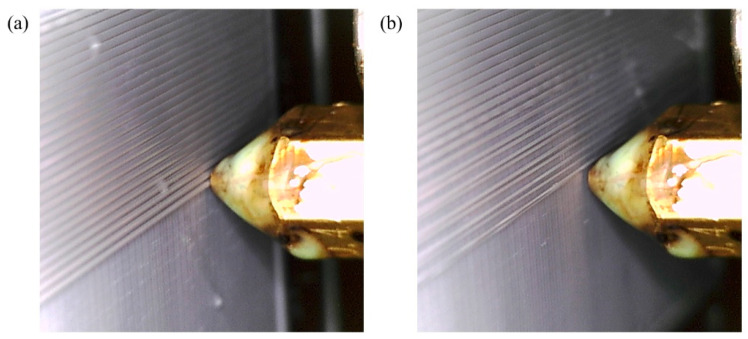
Performance of CLFDCS when a defect due to low temperature occurs. (**a**) Detected defect, (**b**) corrected defect.

**Figure 7:**
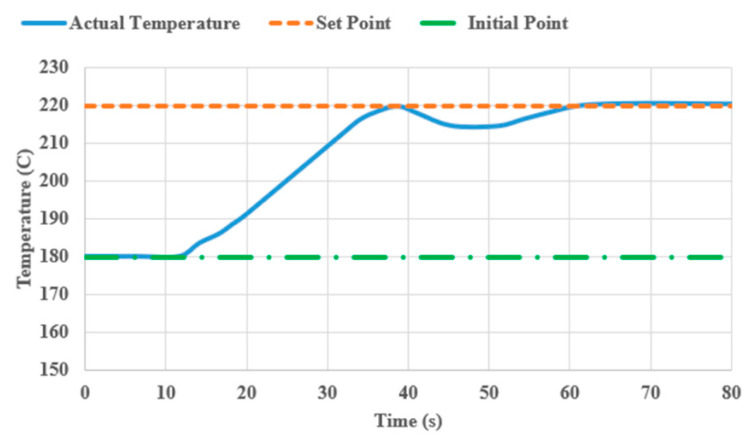
Response time of the CLFDCS with nozzle temperature setting.

Temperature is certainly one of the most crucial print parameters, requiring careful attention to ensure good quality and flawless printed parts. The optimal extrusion temperature for PLA filament typically falls between 200 and 230 °C. While printing at higher temperatures might not always result in a deterioration of surface quality, it can lead to filament damage and adversely affect the mechanical properties of printed parts. Conversely, printing at low temperatures, compared to the recommended range, not only adversely impacts mechanical properties, but also compromises print quality. Thereby, to evaluate the efficacy of our CLFDCS, we conducted a test by printing the first two layers of a plate measuring 70 × 70 × 1.6 mm^3^ at a nozzle temperature of 220 °C. Then, we intentionally lowered the nozzle temperature to 180 °C (β = 40) in the middle of the third layer and activated the CLFDCS. Figure 6a shows a defect resulting from low extrusion temperature, captured by the IAFDM, and promptly corrected by the FCM in Figure 6b. The time response of our proposed system, from activation to surface quality restoration, is shown in Figure 7.

### 4.2. Flow Rate

**Figure 8:**
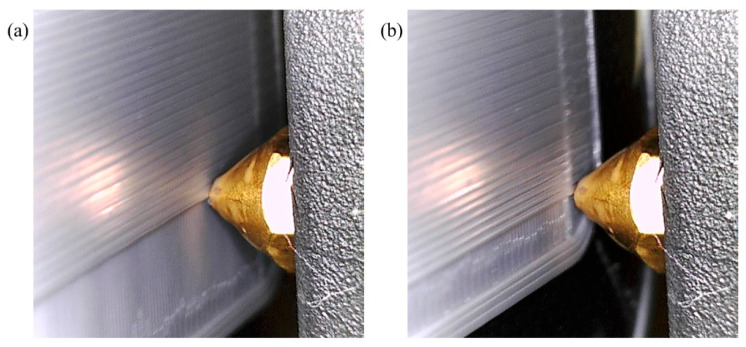
Performance of CLFDCS when a defect due to overflow occurs. (**a**) Detected defect, (**b**) corrected defect.

**Figure 9:**
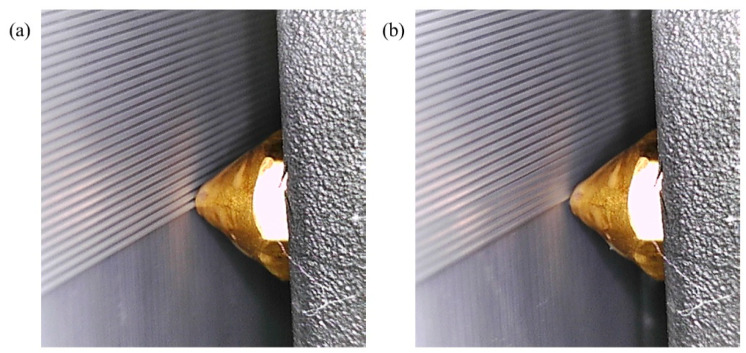
Performance of CLFDCS when a defect due to underflow occurs. (**a**) Detected defect, (**b**) corrected defect.

The system’s efficacy and robustness are demonstrated through rigorous testing that replicates various anomalies in extrusion rates. By deliberately adjusting the flow rates at both low (60%) and high (140%) levels (β = 40), the system adeptly identifies and corrects these irregularities in real-time, highlighting its control capabilities. Visual validation of the system’s operational performance under different extrusion rate conditions is provided in Figure 8 and Figure 9. In Figure 8, the IAFDM successfully identifies an overfill defect resulting from a high extrusion rate, with FCM promptly correcting the flow rates, effectively addressing the defect as shown in the image.

Similarly, Figure 9 displays the system’s ability to detect an underfill caused by a low extrusion rate, promptly readjusting the flow rate to restore the print quality. These visual representations underscore the system’s ability to detect and autonomously resolve extrusion irregularities, ensuring consistent and high print quality throughout the manufacturing process.

**Figure 10:**
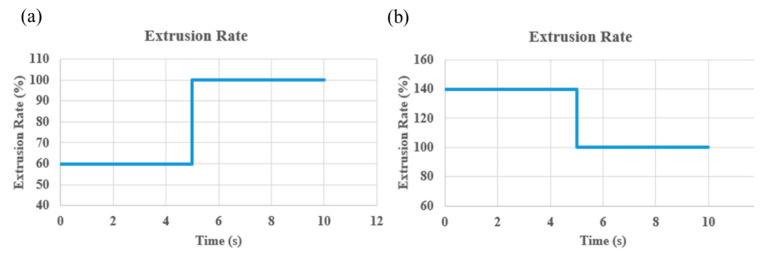
Response time of CLFDCS. (**a**) Response to under extrusion. (**b**) Response to over extrusion.

The response times of the CLFDCS for under extrusion and over extrusion scenarios are shown in Figure 10a,b, respectively. It is evident that the system demonstrates rapid response times in both cases, typically detecting surface errors and adjusting the corresponding print parameter (extrusion rate in this case) within approximately 5 s, before transmitting the modified value to the printer.

### 4.3. Print Speed

**Figure 11:**
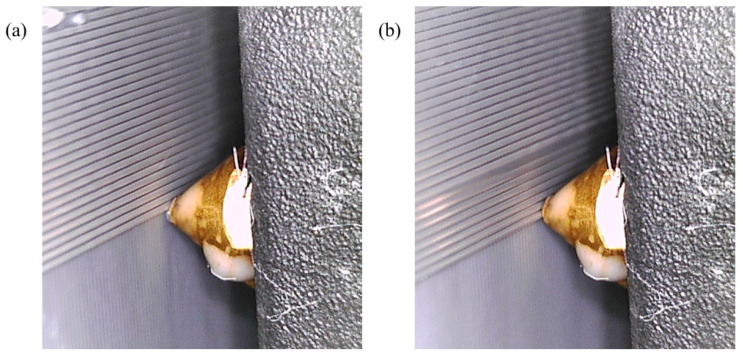
Performance of CLFDCS when a defect due to high printing speed occurs. (**a**) Detected defect, (**b**) corrected defect.

Another print parameter that may affect the print quality in FDM process is print speed. Similar to previous experiments, we printed the first two layers at a standard print speed of 20 mm/s and manually increased the print speed to 150 mm/s (γ = 20/150 = 0.13) in the middle of the third layer in the G-code. After a surface defect occurred due to the high print speed as shown in Figure 11a, CLFDCS was activated. The results of quality recovery, achieved by reducing the print speed back to 20 mm/s, are shown in Figure 11b.

**Figure 12:**
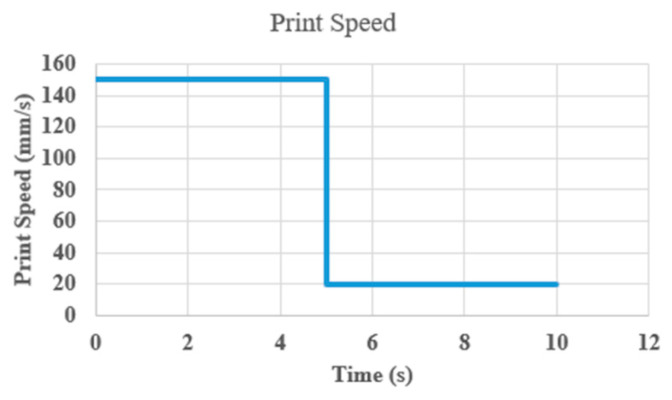
Response time of CLFDCS to high nozzle speed.

The response time of the CLFDCS to a defect caused by high print speed is shown in Figure 12. Following the occurrence of a surface defect due to excessive print speed, as illustrated in Figure 12, the CLFDCS was activated, leading to quality recovery achieved by reducing the print speed back to 20 mm/s.

## 5. Limitations, Improvements, and Future Work

### 5.1. Limitations and Improvements

While this paper explored a novel approach to developing a cost-effective and accessible defect detection and correction system in FDM, numerous avenues for future research to enhance the system’s capabilities exist. One crucial area for improvement involves integrating a PID controller into the correction software. While the current system uses hardcoded values for adjusting print parameters, a PID controller could dynamically adjust these values based on the severity of the detected defect.

Another avenue for improvement is the integration of a thermal camera to monitor the extrusion nozzle temperature and the top-layer print temperature. This would enable accurate temperature monitoring for each layer, aiding in mitigating poor layer adhesion or detecting temperature fluctuations in the nozzle. While particularly useful for FDM printing, this implementation may be more suited for metal additive manufacturing and higher temperature printing due to their temperature requirements and gradients.

Although metal additive manufacturing represents the natural progression for this closed-loop controller, implementing our developed system in this context poses challenges due to differences in deposition type and printing parameter complexity. Consequently, the near-term expansion of this project is more likely to involve the utilization of metal-infused PLA materials, such as readily available aluminum-infused PLAs. These materials print at higher temperatures and enhance part density, facilitating easier void detection implementation.

One major drawback of the current system is its inability to identify internal defects. Possible solutions include implementing in situ acoustic or X-ray scanning to detect porosity and voids or utilizing piezoelectric sensors for detecting abnormalities within the print. While correcting such defects may not be feasible at this stage, this information could be used to halt the print process and conserve material.

Furthermore, enhancing defect detection capabilities by adding extra microscopes poses challenges such as increased tool head weight, potentially affecting printing speed and causing vibrations that could impact print quality and defect detection. To address these challenges, future iterations of the system could incorporate compact designs using lightweight materials like carbon fiber composites or PEEK (Polyether Ether Ketone) 3D printing polymers to minimize additional weight without compromising structural integrity. Implementing vibration dampers could also reduce the impact of nozzle movements on image stability, ensuring consistent defect detection. Additionally, smaller microscopes and high-speed cameras could be integrated or embedded into the printer head to reduce weight and ensure clear imaging at high speeds, facilitating easier mounting and adjustment solutions.

### 5.2. Future Work

In order to enhance the project’s viability, additional testing scenarios will be carried out in the future. This includes considering a broader range of defect variations in various orientations and exploring more complex geometries. The primary focus will be on the standard test coupon geometry. Following printing, the tensile properties of the prints will undergo physical testing to assess the effectiveness of the defect correction system in mitigating defects. This comparative analysis against an ideal print will provide a quantitative representation of the CLFCDS system’s capabilities.

## 6. Conclusions

In this study, we developed a closed-loop fault diagnosis-correction system (CLFDCS) using an Ender 5 Plus FDM printer to address the quality monitoring challenges associated with FDM printing. The CLFDCS integrates digital microscopes for image collection, an image analysis-based fault diagnosis module, and a Fault Correction Module to detect and rectify defects in real-time. The experimental results demonstrate the effectiveness of the CLFDCS in detecting and correcting surface defects caused by variations in temperature, flow rate, and print speed. By intentionally inducing defects and assessing the system’s response, we validated its ability to maintain high print quality across diverse printing conditions. Our approach incorporates an innovative feature extraction method using image textural analysis to identify and extract relevant features from input images for defect recognition. This feature extraction technique proved highly effective in detecting defects, surpassing traditional benchmark methods.

The core strength of our system lies in its real-time adjustment of machine parameters to mitigate detected defects during the printing process. Through automated parameter adjustments, our closed-loop control framework demonstrated significant efficacy in addressing defects and improving overall print quality.

Overall, the CLFDCS represents a promising advancement in enhancing the quality and reliability of FDM-printed parts, with implications for improving manufacturing processes in various industries. Future research may focus on further refining the system’s algorithms and expanding its application to other 3D printing techniques and materials.

## Figures and Tables

**Table 1:** Implementation cost breakdown.

Item	Function	Cost (Est.)	Qty.
Digital microscope	Collect images for defect detection	USD 150	2
USB-C cable	Connect the computer to the printer’s serial port	USD 20	1
Hardware package	Used to install the microscope brackets	USD <10	1
PLA (1 kg roll)	Used to print microscope backets	USD 25	1

**Table 2:** Fault Correction Module—conditions, actions, and effects.

I_1	I_2	Type of Defect	Action	Effect
0	0	Normal	No action needed.	G-Code remains unchanged.
1	1	Overfill-Flow rate	Sends single command: M221 S(Current Value − α); Initializes I_1 and I_2 to 0.	Reduces flow rate by α to mitigate overfill defect.
2	2	Underfill-Flow rate	Sends single command: M221 S(Current value + α); Initializes I_1 and I_2 to 0.	Increases flow rate by α to mitigate underfill defect.
	3	Underfill-Temperature	Sends single command: M104 S(Current Value + β); Initializes I_1 and I_2 to 0.	Increases nozzle temperature by β degrees to mitigate underfill defect.
	4	Underfill-Speed	Changes F multiplier from 1 to γ (γ < 1).	Multiplies all values of F by γ to decrease movement speed to mitigate underfill defect.

## Data Availability

Data are contained within the article.
